# Pregnancy, fertile life factors, and associated clinical course in *PRKN* early-onset Parkinson’s disease

**DOI:** 10.1007/s10072-023-07029-8

**Published:** 2023-09-02

**Authors:** Roberta Bovenzi, Matteo Conti, Giulia Rebecca Degoli, Rocco Cerroni, Carlo Alberto Artusi, Mariangela Pierantozzi, Alessandro Stefani, Nicola Biagio Mercuri, Tommaso Schirinzi

**Affiliations:** 1 Unit of Neurology, Department of Systems Medicine, University of Rome “Tor Vergata”, Via Montpellier, 00133 Rome, Italy; 2 Department of Neuroscience “Rita Levi Montalcini”, University of Turin, Turin, Italy; 3 UOSD Parkinson Centre, Tor Vergata University Hospital, Rome, Italy

**Keywords:** Parkin, Early onset Parkinson’s disease, Pregnancy, Fertile life, Sex hormones

## Abstract

**Introduction:**

As the most common cause of autosomal recessive early onset Parkinson’s disease (EOPD), parkin type Parkinson’s disease (*PRKN*-PD) may affect female patients in childbearing age. Accordingly, issues related to fertility must be adequately addressed. Here, we landscaped fertile life factors and pregnancy course of a *PRKN*-PD cohort, including both novel cases directly observed at our center and published ones.

**Methods:**

Six patients with confirmed *PRKN-*PD were examined by a structured interview on reproductive factors and associated modifications of PD disturbances, including one case followed up throughout pregnancy which was described in greater detail. Six studies reporting fertile life factors of nine PRKN-PD patients were reviewed collecting homogeneous data on fertile life and pregnancy course.

**Results:**

*PRKN*-PD female patients experienced motor fluctuations with the menstrual cycle, pregnancy, and puerperium, which suggests a role for sex hormones in PD clinical burden. In some cases, abortion and miscarriages occurred during the organogenesis phase in patients receiving oral antiparkinsonian therapy; however, levodopa/benserazide monotherapy resulted to be the safest choice in pregnancy.

**Conclusion:**

Collectively these data disclose the importance of pre-conception counseling in childbearing age *PRKN*-PD patients and EOPD in general.

## Introduction

Parkinson’s disease (PD) is a neurodegenerative disorder due to the loss of dopaminergic nigral cells and the brain accumulation of α-synuclein-positive Lewy bodies [1].

Although PD most commonly occurs in the elderly, in about 10% of cases, it affects subjects younger than fifty, presenting as “early-onset PD” (EOPD) [2–5].

PD impacts patients differently depending on their age at onset [2]. In particular, females with EOPD face peculiar challenges due to fertile life, including menstruation, pregnancy, and breastfeeding [6].

A genetic origin is almost frequent in EOPD [7]. Namely, bi-allelic mutations in the *PRKN* (Parkin) gene account for the most common autosomal recessive form of PD, representing about 15–20% of EOPD cases [8]. *PRKN* type PD (*PRKN*-PD) patients exhibit unique features compared to idiopathic EOPD patients, such as more prevalent dystonia at onset, sleep benefit, lower incidence of postural instability and falls, and better clinical course overall, including lower rate of cognitive impairment [9].

Because of the relative frequency of *PRKN*-PD among EOPD patients, issues related to fertile female life and pregnancy must be adequately and specifically addressed.

In this study, we examined fertile life factors and pregnancy courses in a cohort of *PRKN*-PD females, including six cases directly observed at our center and a group of previously described patients, to outline the PD course along the fertile life steps and identify helpful clues for clinical management.

## Methods

### Novel case series

Six confirmed *PRKN*-PD female patients from the Neurology Unit of the Tor Vergata University of Rome (Italy) were systematically assessed for fertile life history through a structured interview. One case followed up throughout pregnancy was described in greater detail. The questionnaire was structured into two parts: the first focused on general fertile life factors (age at menarche, regularity of menstruations, modification of symptoms during menstruations, number of pregnancies, type of births, number of abortions, age at menopause); the second, if applicable, focused on the pregnancy course after PD diagnosis (number of pregnancies, modification of symptoms and PD medications during pregnancies, type of births, number and type of abortions, information about breastfeeding). Informed consent was obtained from each participant. The study was conducted by the principles of the Helsinki Declarations and approved by the local ethical committee (protocol number 120/21).

### Literature review

An in-depth literature search of the PubMed database using the Systematic Review Tool/Rayyan was performed to collect all patients with *PRKN*-PD previously described for fertile life factors [10]. The final reference list was generated after selecting all the manuscripts with key information regarding the selected topics: “Parkinson’s disease,” “*PRKN*,” “Parkin,” “*PARK2*” plus “pregnancy,” “menstruations,” “menstrual cycle,” “menses,” “breastfeeding.” A total of 192 articles were screened for relevant content (including duplicates). Of these, 22 were selected for further review. A final reference list of six articles, excluding duplicates, was finally included in this study, providing a description of fertile life factors and/or pregnancy courses in nine *PRKN*-PD patients [11–16]. As for the local cohort, we collected data on genetics, motor fluctuations during menstruation, pregnancy (including clinical course, therapies, and outcome of pregnancy), and breastfeeding.

## Results

### Novel case series

#### Case 1

A woman carrying a compound heterozygous mutation in the *PRKN* gene (exon 3 deletion in one allele and *PRKN* c1285 + 1G > A variant in the other allele) had been in our care since the age of 30 when she presented with asymmetrical bilateral lower limb dystonia, mild right upper limb bradykinesia, and rigidity, consistent with PD diagnosis (positive DAT-spect and ascertained levodopa 150 mg response). She was treated for a long time with pramipexole (0.52 mg/day), rasagiline (1 mg/day), and trihexyphenidyl (2 mg/day), reporting a full symptomatic control except for some motor and non-motor fluctuations (anxiety and depressed mood) during the menstrual cycle, with worsening a few days before menstruations and improvement afterward. After the patient expressed a desire for pregnancy at the age of 36, genetic counseling for the couple was performed. Because of the insufficient data on pregnancy safety, rasagiline, pramipexole, and trihexyphenidyl were discontinued, shifting to single therapy with levodopa/benserazide (300 mg/75 mg/day). The couple experienced difficulties conceiving due to gynecological troubles (a damaged tube) and the partner’s poor sperm motility. Accordingly, two cycles of medically assisted procreation (in vitro fertilization, IVF) were attempted, during which the patient did not report any modification in PD symptoms associated with exogenous hormonal treatment. At the age of 40, the patient had a first-trimester miscarriage due to an anembryonic pregnancy. Later, at the age of 41, she became pregnant again. This pregnancy had a normal course, and the levodopa/benserazide therapy was safely maintained at the same dosage (300 mg/75 mg/day). In the first trimester, the patient complained of fatigue and asthenia; then, both the motor disturbances and the general perception of well-being improved. At week 40 of gestation, the patient gave birth to a healthy baby girl through vaginal delivery. Immediately after delivery, the patient experienced a profound worsening of motor and non-motor symptoms. Breastfeeding was avoided to prevent drug passage, so safinamide was introduced (up to 100 mg/day) as an add-on, leading to stable clinical improvement. The baby, at the age of 6 months, was perfectly normally developing.

#### *PRKN*-PD local cohort

Table 1 summarizes the main fertile life and pregnancy features together with related PD issues of the six-*PRKN*-PD patient cohort observed at our center, including case 1 and the other five unpublished ones. The mean AAO was 35.2 ± 4.7 (30–40) years. Four patients (66.7%) reported worsening of both motor and non-motor disturbances a few days before menses. One of these noticed an amelioration of such menstrual cycle-related fluctuations after estrogen replacement therapy. Four patients got pregnant, for a total of ten pregnancies. Of these, seven resulted in live births (70%), two were electively terminated (20%), and one resulted in spontaneous abortion (case 1, 10%). Delivery was vaginal in four cases and by cesarean section (for reasons unrelated to PD) in three cases. Case 1, the only pregnant woman after the PD diagnosis and taking PD medications, reported motor fluctuations during pregnancy and worsening after delivery. Levodopa/benserazide treatment was kept unchanged during all the pregnancy course without complications. Of interest, case 2 experienced transient rigidity and pain in the left lower limb during both the first pregnancy (before PD diagnosis) and 2 years later (a few months after the second pregnancy), when she was diagnosed with PD. No cases of restless leg syndrome (RLS) were diagnosed. Case 1 avoided breastfeeding. Case 2 could not breastfeed for reasons unrelated to PD.

**Table 1:** Main fertile life factors and pregnancy features of the local *PRKN*-PD patients cohort

Patient	Genetics	AAO (years)	Symptoms/management during menstruations	Number of pregnancies	Description of pregnancies	Breastfeeding
Case 1	*PRKN* exon 3 deletion het + *PRKN* c1285 + 1G > A het pathogenic variant	30	Worsening of motor and non-motor features before menses	*N* = 2, medical assisted, after PD diagnosis	*N* = 1 early miscarriage due to anembryonic pregnancy, no changes in PD medications (levodopa/benserazide 300/75 mg/day) *N* = 1 full-term pregnancy, vaginal birth, healthy baby girl. Perception of asthenia/fatigue during the first trimester of pregnancy, rapidly ameliorated during the second and third semester. No changes in medications (levodopa/benserazide 300/75 mg/day)	No, artificial milk to avoid exposure of PD medications
Case 2	*PRKN* exon 3 deletion homo	30	Worsening of motor and non-motor features before menses	*N* = 3, before PD diagnosis	*N* = 2 full-term pregnancies, C-section, 2 healthy babies Pain and rigidity in right lower limb in the third trimester during the first pregnancy and in the puerperium of the second pregnancy *N* = 1 voluntary abortion	No, artificial milk for reasons unrelated to PD
Case 3	*PRKN* exon 5,6 duplication	40	Worsening of motor and non-motor features before menses, response to estrogenic replacement treatment after menopause	*N* = 3, before PD diagnosis	*N* = 2 full-term pregnancies, 1 vaginal birth, and 1 C-section, healthy babies *N* = 1 miscarriage	Yes
Case 4	*PRKN* exon 3,4 deletion	33	Worsening of motor and non-motor features before menses	*N* = 0	NA	NA
Case 5	*PRKN mutation, NS*	39	No modifications	*N* = 0	NA	NA
Case 6	*PRKN* exon 5.6 deletion	39	No modifications	*N* = 2, before PD diagnosis	*N* = 2 full-term pregnancies, vaginal births, healthy babies	Yes

*AAO*, age at onset; *Het*, heterozygous; *homo*, homozygous, *NA*, not applicable, *NS*, not specified

### Literature review

Table 2 reports the main demographic and clinical features of patients. The onset of symptoms was defined in seven patients, with a mean AAO of 25.1 ± 9.9 (12–37) years. Three patients reported menstrual-related motor worsening, which improved in two cases with exogenous estrogenic treatment. Seven patients had a pregnancy history, for a total of 18 pregnancies. Seventeen pregnancies occurred after PD diagnosis, all in patients receiving oral antiparkinsonian therapy. Of those, 61.1% (*n* = 11) resulted in live births, 22.2% (*n* = 4) were electively terminated due to concerns about antiparkinsonian drugs, and 16.7% (*n* = 3) resulted in spontaneous abortion. The antiparkinsonian therapy was maintained during pregnancy in 15 cases, with 53.3% (*n* = 8) resulting in live births and the others resulting in spontaneous or voluntary abortion. Three patients became pregnant while on STN-DBS stimulation. Three of the seven pregnant patients (42.9%) complained of a worsening of their motor symptoms during pregnancy or soon after delivery; one patient (14.3%) ameliorated but had an early spontaneous abortion. No RLS cases were diagnosed. C-sections were performed in two patients because of the premature rupture of the membranes in a twin pregnancy in one case and because of the abnormal fetus position in the other. Two of the twelve newborns (16.7%) had a cardiac malformation. Three patients avoided breastfeeding, and two breastfed without taking any oral medication (one with STN-DBS). Lactation information was missing in the other cases.

**Table 2:** Main fertile life factors and pregnancy features of the literature rewiev *PRKN*-PD patients cohort

Study	Genetics	AAO (years)	Symptoms/management during menstruations	Number of pregnancies	Description of pregnancies	Breastfeeding
Serikawa et al., 2011 (*N* = 1)	*PRKN* exon 2–4 deletion	12	Worsening of symptoms between ovulation and menstruation	*N* = 1 dichorionic/diamniotic twin pregnancy, after PD diagnosis	Inpatient hospital care and discontinuation of antiparkinsonian drugs except levodopa/carbidopa (450 mg/day); worsening of motor symptoms in the third trimester; gradually increase levodopa/carbidopa up to 700 mg/day, resumption of entacapone (400 mg/day) and selegiline (7.5 mg/day) C-section at 35th week of gestation due to preterm premature rupture of the membranes Two healthy male twins, non-surgical ventricular septum defect in one of the babies	No, synthetic milk to avoid exposure to PD medications
Sprenger et al., 2014 (*N* = 2)	Patient 1: genetic testing NA (sibling of patient 2) Patient 2: *PRKN* intron 6 homo pathogenic variants (c.734 + 1G > A)	34 37	Patient 1,2: perimenstrual worsening of symptoms and response to dienogest-ethinylestradiol (75 lg) for continuous use	NA	NA	NA
Scelza et al., 2015 (*N* = 3)	Patient 1, NS “*Parkin* mutation”	19	NS	*N* = 4, after PD diagnosis	*N* = 1 healthy female, NS birth, NS PD medications *N* = 1 female with suffering from a severe atrial defect, NS birth, NS PD medications *N* = 1 voluntary abortion (fear of teratogenic effects of antiparkinsonian drugs) *N* = 1 single pregnancy while on STN-DBS, pharmacological treatment (NS) discontinuation without worsening of motor symptoms; C-section at full term because of the baby’s abnormal position; worsening of right leg dystonia and impairment in walking 1 week after delivery	*N* = 2 NS (prior to STN-DBS) *N* = 1 safe breastfeeding (while on STN-DBS, no pharmacological treatment)
	Patient 2 and patient 3, NS “Parkin mutations”	NS	NS	Patient 2,3 *N* = 4 singleton pregnancies, after PD diagnosis	Patient 2,3, *N* = 2 “healthy pregnancies,” while on STN-DBS + PD NS pharmacological treatment. Type of birth and symptoms NS Patient 2, *N* = 2 voluntary abortions (fear of teratogenic effects of PD medications) Patient 3, *N* = 2 spontaneous abortion for unknown reasons (the first when taking levodopa, the second when taking rasagiline, trihexyphenidyl, and levodopa)	Patients 2 and 3, no breastfeeding but synthetic milk to avoid exposure to PD medications
Sazci, 2019 (*N* = 1)	*PRKN* three silent mutations: bases 429 (C > T), 513 (G > A), and 667 (C > T). The missense mutations detected at bases 932 (A > G) and 1111 (G > A) replace Gln311 with Arg and Ala371 with Thr respectively	34	NS	*N* = 1 singleton pregnancy before PD diagnosis *N* = 1 singleton pregnancy after PD diagnosis	*N* = 1 “Healthy baby,” type of birth NS (before PD diagnosis) *N* = 1 miscarriage at first semester (after PD diagnosis), no changes in PD medications: levodopa 100 mg/carbidopa 25 mg/200 mg × 5/day, pramipexole 1 mg × 3/day, rasagiline 1 mg/day. Amelioration of motor symptoms (H&Y from stage 3 to stage 1, MDS-UPDRS part III from 32 to 6)	NS
Lim et al., 2021 (*N* = 1)	*PRKN* homozygous p.Cys441Arg (c.1321 T > C)	16	NS	*N* = 1 singleton pregnancies after PD diagnosis *N* = 1 voluntary abortion after PD diagnosis (fear of teratogenic effects of PD medications)	*N* = 1 “Healthy boy,” type of birth NS, levodopa, symptoms NS	NS
Fumihito Yoshii et al., 2021 (*N* = 1)	*PRKN* exon 3–4 deletion, c.535-3A > G	24	NS	*N* = 1 singleton pregnancy after PD diagnosis	*N* = 1 healthy baby, type of birth NS, levodopa discontinuation with worsening of lower limbs dystonia	Safe breastfeeding, no pharmacological treatment, resumption of levodopa/carbidopa after breastfeeding (200 mg/die) with benefit on lower limbs dystonia

*AAO*, age at onset; *het*, heterozygous; *homo*, homozygous; *NA*, not applicable; *NS*, not specified

## Discussion

This study, combining data from both the local cohort and previously described cases, provided an unprecedented overview of issues related to fertile female life and pregnancy in *PRKN*-PD, a common form of EOPD.

First, we examined the impact of the menstrual cycle on the main PD disturbances and found that most *PRKN*-PD patients experienced a worsening of motor and non-motor symptoms immediately before menstruation [12], which is consistent with previous reports on idiopathic PD [17–20]. Of interest, the subjects by Sprenger et al. and one of our cases improved in menstruation-related fluctuations with the introduction of hormonal replacement therapy, suggesting that clinical deterioration before menses could result from the nadir of sex hormones [12]. However, other general factors due to the premenstrual global function and feeling of well-being might play a role [21].

Fertility issues are still poorly explored in PD female patients [22]. Our case 1 required medically assisted pregnancy because of the couple’s infertility due to gynecological factors (a damaged tube) rather than PD-related factors. The IVF was successful, as described in a previous case [23].

About 14% of the *PRKN*-PD patients’ pregnancies resulted in spontaneous abortion and about 20% in voluntary interruption. This abortion rate matches that of the general population (estimated to range from 10 to 24% [24]) but seems higher than that reported for idiopathic PD patients [17]. Two of the four miscarriages occurred in patients taking antiparkinsonian medications. As well, all cases of voluntary abortion were motivated by concerns about the teratogenic effects of PD medications. Finally, about 17% of the newborns, all exposed to antiparkinsonian medications during pregnancy, had a cardiac defect. Indeed, the effects of movement disorders medications on the fetus are largely unknown [25, 26]. Levodopa monotherapy has been reported as safe in pregnancy [24, 27], while data on dopamine agonists, MAO-B and COMT inhibitors, and anticholinergics are scarce [24]. Amantadine, instead, might have teratogenic effects [28]. Thus, pre-conception multidisciplinary counseling emerges as fundamental to providing education and maximizing pregnancy outcomes in all patients of childbearing age [29].

Eighteen pregnancies resulted in live births. Eleven pregnancies occurred after PD diagnosis, with nine patients taking oral medications. Five of eight patients (62.5%) who became pregnant after being diagnosed with PD reported fluctuations of motor features associated with pregnancy or puerperium, namely improvement during pregnancy in two cases (including our case 1), worsening during pregnancy in two cases, and worsening after delivery in two cases (including our case 1). Such fluctuations can be essentially related to hormonal changes, since estrogen and progesterone levels rise during pregnancy and dramatically drop with puerperium. While the neuroprotective and pro-dopaminergic effects of estradiol (E2) are well-known [30], the properties of estriol (E3), the dominant estrogen during pregnancy, and progesterone are not clear yet [17]. Apart from sex hormones, other factors, including altered pharmacokinetics, diet changes, and variations in intestinal absorption, as well as physical and psychosocial aspects, might also contribute to motor fluctuations during pregnancy [17]. The existing literature on pregnancy-related motor fluctuations is not univocal, although in most cases, a clinical worsening has been described either in pregnancy or in puerperium [17, 27], often due to medication reduction or withdrawal [3]. Indeed, the two patients who improved with pregnancy had been on the same oral medication since conceiving (levodopa monotherapy in one case, levodopa, pramipexole, and rasagiline in the other one). Patients who clinically deteriorated, on the other hand, had reduced or discontinued oral therapy, emphasizing the importance of the proper medication and dosage to ensure the patient’s well-being during such a critical phase.

It is interesting to notice that case 2 most likely experienced prodromal motor manifestations (as foot dystonia) during her first pregnancy and the puerperium of her second pregnancy, when an overt PD was recognized. Dystonia, indeed, may represent an onset sign of PD, especially in *PRKN*-PD [31, 32], although some cases of “dystonia gravidarum,” in which dystonia appears in pregnancy and rapidly resolves after delivery, have already been described [33, 34].

Of all live birth pregnancies reported, five resulted in a cesarean Sect. (5/18, 27.7%), reflecting the rates of C-sections of the general population (33.7% in Italy) [35] and, again, indicating no excess rates in this group population.

Breastfeeding was practiced only in two cases, both in the absence of oral medications. The benefits of breastfeeding either for the newborn or the mother are well established [36]; however, there is not sufficient evidence for antiparkinsonian agents’ safety in breastfeeding [28]. Levodopa, dopamine agonists, amantadine, and entacapone can indeed be detected in breast milk and may potentially affect the infant [28]. Nevertheless, some patients taking low doses of levodopa/carbidopa practiced breastfeeding without apparent harm on the infant [37].

## Conclusion

The analysis of this small *PRKN*-PD cohort allowed us to highlight the impact of fertile female life on PD clinical course and, more in general, provide some insightful cues for the management of EOPD in women.

We reported how levodopa/benserazide monotherapy could assure a safe pregnancy (IVF-mediated) in PD without worsening of clinical impairment. Then, we noticed a rate of voluntary abortion and miscarriages during organogenesis in orally treated *PRKN*-PD patients, which raises the need for pre-conception counseling in young patients of childbearing age.

Finally, we observed that *PRKN*-PD presents with motor fluctuations concurring with the menstrual cycle, pregnancy, and puerperium, which definitely emphasizes the contribution of sex hormones in PD clinical burden, also paving the way for novel, potential therapeutic interventions [38].

## Data Availability

The datasets generated during analysis are available from the corresponding author upon reasonable request.
